# Mesenchymal Stem Cell Derived Extracellular Vesicles for Repairing the Neurovascular Unit after Ischemic Stroke

**DOI:** 10.3390/cells10040767

**Published:** 2021-03-31

**Authors:** Courtney Davis, Sean I. Savitz, Nikunj Satani

**Affiliations:** Institute for Stroke and Cerebrovascular Diseases, McGovern Medical School at UTHealth, Houston, TX 77030, USA; Courtney.Davis@uth.tmc.edu (C.D.); Sean.I.Savitz@uth.tmc.edu (S.I.S.)

**Keywords:** preconditioning, blood-brain barrier, bioengineering, hypoxia, ischemic stroke, cell therapy, secretome, personalized treatment

## Abstract

Ischemic stroke is a debilitating disease and one of the leading causes of long-term disability. During the early phase after ischemic stroke, the blood-brain barrier (BBB) exhibits increased permeability and disruption, leading to an influx of immune cells and inflammatory molecules that exacerbate the damage to the brain tissue. Mesenchymal stem cells have been investigated as a promising therapy to improve the recovery after ischemic stroke. The therapeutic effects imparted by MSCs are mostly paracrine. Recently, the role of extracellular vesicles released by these MSCs have been studied as possible carriers of information to the brain. This review focuses on the potential of MSC derived EVs to repair the components of the neurovascular unit (NVU) controlling the BBB, in order to promote overall recovery from stroke. Here, we review the techniques for increasing the effectiveness of MSC-based therapeutics, such as improved homing capabilities, bioengineering protein expression, modified culture conditions, and customizing the contents of EVs. Combining multiple techniques targeting NVU repair may provide the basis for improved future stroke treatment paradigms.

## 1. Introduction

Stroke is one of the leading causes of mortality and long-term disability in the United States, accounting for approximately 1 out of every 19 deaths and 2.4% of adult disability [1,2]. Worldwide in 2019, approximately 6.6 million deaths could be attributed to stroke [1,3]. A stroke occurs when blood flow to the brain is impaired. Ischemic stroke comprises 87% of all stroke cases and is caused by an artery obstruction interrupting blood delivery [1,4]. The reduction in blood flow, which supplies nutrients to the brain, initiates a series of interconnected cascades that cause widespread tissue damage and death [5]. The blood-brain barrier (BBB) impairment is one of the main pathologies associated with ischemic stroke. BBB insult begins early, before the onset of neuronal damage and influences the extent of brain injury [6,7].

Mesenchymal stem cells (MSCs, also known as mesenchymal stromal cells) have been frequently explored as a promising potential therapy for recovery after ischemic stroke. However, since very few MSCs reach the brain, it is believed that most of the therapeutic effects of MSCs are the result of paracrine signaling [8]. One of the likely mediators for such signaling are the extracellular vesicles (EVs) released by MSCs [9,10]. EVs could mediate the transmission of molecules such as lipids, proteins, nucleic acids, cytokines, chemokines, and growth factors that may alter the behavior and phenotype of the receiving cell [11,12]. In this review, we discuss the potential role of EVs after stroke in repairing the BBB. We also discuss strategies to modulate the EV phenotype and secretion to create a more pro-regenerative environment for recovery of BBB after stroke.

## 2. Changes in Blood-Brain Barrier after Stroke

The BBB is an interface that separates the central nervous system from the blood circulating in the periphery. It is a highly selective barrier and is responsible for a number of roles essential for normal brain function, including the maintenance of homeostasis, protection against pathogens and other potentially harmful substances, nutrient transport, and maintaining the optimal physiological/extracellular environment of the brain for neuronal and glial functions. The principle component of the BBB is the endothelial cells (ECs) linked together by tight junctions (TJs) that form the walls of the brain vasculature and microvasculature, however these are only one part of the broader structure that regulates the movement of molecules between the blood and brain. The whole “neurovascular unit” (NVU; or “extended BBB”) is a highly intricate and dynamic network of cellular and non-cellular interplay that regulates BBB permeability and cerebral blood flow [13]. Other components of the NVU include pericytes, neural cells, astrocytes, and microglia [13,14,15]. Deficiencies in any of the components of the NVU may result in a leaky or damaged BBB, which has severe implications on brain health. Such interconnectedness of the NVU means that in vitro models of the BBB require co-culture of multiple cell types in order to replicate even basic barrier properties [16,17].

Within minutes of stroke onset, neurons in the infarct area release massive amounts of glutamate and create an excitotoxic environment. Lack of blood flow does not allow for replenishment of oxygen and removal of cellular byproducts. This leads to the accumulation of toxic substances, such as reactive oxygen species (ROS) and NO, as well as depletion of ATP. Local immune cells such as astrocytes and microglia are activated and produce substances such as matrix metalloproteinase-9 (MMP9) that contribute to the disruption of the TJs holding the BBB together [18,19]. Energy depletion causes dysfunction of transmembrane pumps, rendering neurons unable to maintain ionic balance. Accumulation of intracellular Ca^2+^ and Na^+^ causes cellular swelling and membrane degeneration, allowing dissemination of danger signals [20]. Consequently, hematogenous immune cells and inflammatory factors that are normally blocked by the BBB are able to permeate into the ischemic brain tissue and inflict further injury. Over the subsequent hours and days, the large scale inflammatory response in the brain, abetted by an injured BBB and vascular breakdown, leads to irreversible neuronal death in the infarct region [21,22]. Indeed, neuro-inflammation is a hallmark of stroke and several other neurological disorders, and is a key factor in BBB disruption [5,19].

Whether the increased BBB permeability after stroke is caused by loss of intracellular junctions or by increased transcellular transport through ECs via caveolae and vacuoles is debated, although the variations in these findings may be due to time point differences. Nahirney et al. used electron microscopy to view the peri-infarct endothelium at 3 and 72 h after stroke [23]. They found a significant increase in the number of vacuoles at both time points and very few disrupted TJs, although the number of disruptions did increase with time [23]. Krueger et al. found that TJs appeared intact 25 h after stroke, despite the evidence of BBB leakage [24]. On the other hand, there is ample evidence for the breakdown of junction proteins caused by cerebral ischemia. The phosphorylation of occludin, claudin-5, and ZO-1 is mediated by increased activation of nPKC-θ and aPKC-ζ caused by hypoxia-reoxygenation stress [25]. Pro-inflammatory cytokines and activation of NAPDH oxidase are known to further disrupt the association between ZO-1 and occludin [26]. There is reduction in transcription or increased degradation of TJ proteins after stroke [27]. Additionally, hemorrhagic transformation may be due to TJ disruption. Taken together, the evidence points to the notion that TJ protein degradation contributes to BBB disruption.

Studies point towards a biphasic pattern of BBB permeability and disruption caused by different mechanisms. The first phase occurs approximately 6 h after reperfusion and involves increased permeability through the caveolae-mediated transcytosis of non-selective molecules across ECs [28]. This increase in caveolae activity may be caused by pericyte migration away from the BBB [28,29]. In the second phase, about 48–60 h after reperfusion, the protein junctions between ECs are broken down and remodeled [28]. Studies do not always consider that there may be different phases of BBB permeability, but it is an important aspect of stroke pathology that should be taken into account when determining appropriate therapies.

## 3. Current Stroke Management and Its Limitations

Currently there are only two FDA-approved therapies for stroke, and both require immediate medical care. Intravenously administered tissue plasminogen activator (tPA), a thrombolytic agent, acts to dissolve any blood clots to resume normal blood flow. However, tPA increases the risk of hemorrhagic transformation by enhancing the activity of MMPs that dissolve basal lamina supporting the integrity of the vasculature [30,31]. The short window for which tPA’s therapeutic benefits outweigh the potential negative effects is only about 4 h after symptom onset, a window which is reliant on the integrity of the BBB [31,32,33]. Endovascular mechanical thrombectomy is a therapy that extends the time window for therapeutic intervention after symptom onset and increases rates of successful reperfusion. It is used for severe strokes caused by a large vessel occlusion, but it is not always successful in preventing further damage [34].

Restoring blood flow to the injured area is vital, however the reperfusion of blood after ischemic damage causes inflammatory cascades that may add insult to the area. Reperfusion stimulates leucocyte adhesion, oxidative stress, mitochondrial dysfunction, and inflammation that further aggravates brain tissue and vasculature [19,35,36,37]. Despite this, restoration of blood flow is vital, as late perfusions may exacerbate the damage [30,38,39]. Furthermore, blood vessels—especially microvessels—are at risk of collapse when they lack sufficient blood flow, a phenomenon known as “no-reflow”, where reperfusion fails to occur in individual vessels despite blood supply being restored to the area [40,41]. A key factor in the no-reflow phenomenon is astrocytic damage during the initial phases of ischemia [42,43]. Due to the unbalanced cerebral environment, astrocytes swell, causing their end-feet processes to compress the microvasculature even after cerebral flood flow is restored [42,43]. It has been suggested that the administration of osmotherapeutics targeted at reducing the swelling right before or after thrombolysis may help alleviate secondary focal infarction [42]. Restoring blood flow too early, before the vasculature is sufficiently stabilized, increases the risk of hemorrhagic transformation, which may lead to death [44]. Despite the risks, early reperfusion is associated with reduced mortality and greater functional recovery [45].

Stroke presents a notoriously complex pathology, and different therapies may produce different outcomes, depending on when they are administered throughout the progression of the disease. The goals of an ideal stroke therapy would be to restore blood flow to the hypoperfused penumbral region, reduce the size of the infarct, boost regeneration in the afflicted area, and promote functional recovery through reorganization of vascular and neural networks. Thrombolysis and thrombectomy achieve the first goal. Much research has been focused on therapies targeted towards protection and regeneration of parenchymal neurons and glia, with less attention on the recovery of the vital supportive cells and tissues upon which the functional parenchymal cells rely. Indeed, proper neurological function is intimately coupled with NVU health and repair [45]. Hence, we discuss the repair and restoration of the NVU evoked by MSC therapy and their extracellular vesicles (MSC-EVs).

## 4. Extracellular Vesicles from Mesenchymal Stromal Cells

MSCs have been frequently explored as a promising potential therapy for ischemic stroke. Large and readily harvested quantities of MSCs can be derived from bone marrow and adipose tissue, though they can be derived from other tissues as well [46,47]. Very few MSCs administered after stroke actually reach the infarct area. Instead, it is believed that most of the therapeutic effects of MSCs are the result of paracrine signaling [8]. Paracrine signaling can be mediated by releasing trophic factors via extracellular vesicles (EVs) [8,9,10]. Cargo carried by EVs such as lipids, proteins, nucleic acids, cytokines, chemokines, and growth factors can alter the behavior and phenotype of the receiving cell [11,12].

EVs are classified into four types, based on their size and genesis, though all arise from the cell’s plasma membrane: Exosomes, Microvesicles (MVs), Apoptotic bodies, and Oncosomes. The smallest of the EVs are called exosomes. They are 50nm–150nm in diameter and are formed when multivesicular endosomes (MVEs) in the interior of a cell fuse with the outer plasma membrane and dump their cargo into the extracellular space [11,48,49,50]. Microvesicles (MVs) typically range from 50nm–500nm but can be as large as 1000 nm in diameter. These EVs are shed by the outward budding and subsequent cleavage of the cell membrane [11,48,49,50]. Apoptotic bodies are formed by the membrane blebbing of an injured cell as it undergoes apoptosis. They range from 100nm–5um [50,51]. Oncosomes are formed from the irregular membrane protrusions on malignant tumors, and may range from 1um–10um in size [11,50]. Only exosomes and MVs are relevant to the therapeutic effects imparted by MSC-EVs. A summary of different characteristics for subtypes of EVs are described in Table 1 below [11,48,50,51,52,53,54,55,56].

Angiogenesis is part of the brain’s endogenous repair process after ischemic injury. Recovery of the cerebral vasculature and neuronal recovery are tightly coupled [57,58]. Ischemic stroke patients with greater angiogenesis and vasculogenesis have longer survival times, while older patients with reduced new vessel formation fare worse [59,60]. In addition, post-stroke dementia may be related to lower cerebral perfusion and impairments of the NVU [61,62]. Research suggests that administration of MSCs and MSC-EVs is able to boost the brain’s regenerative potential [63,64,65,66,67]. The therapeutic effects of MSC-EV administration yield functionally equivalent benefits to MSC administration, including angiogenesis, neuroprotection, neurogenesis, and functional recovery [63,64,65]. Critically, MSC-EVs are able to go one step farther than MSCs; they can cross the BBB. [68].

MSC-EV administration attenuates post-ischemia immunosuppression, resulting in an environment favorable to neuronal recovery [63]. In a rat traumatic brain injury (TBI) model, MSC-derived exosomes did not affect lesion volume; however; it did improve functional recovery, increase vascular density, increase the number of new neuroblasts, reduce inflammation, and increase angiogenesis [69]. Administration of MSC-EVs during the subacute phase of neonatal hypoxic-ischemic (HI) brain injury resulted in increased proliferation of endothelial cells, as well as a reduction in pro-inflammatory astroglia and microglia activations [66]. These studies show that MSC-EVs exert positive regenerative effects on the ruptured BBB.

## 5. Factors Contributing to Paracrine Benefits of MSCs and Its EVs in Ischemic Stroke

MSC-EVs can carry a huge cargo of beneficial factors, which can contribute positively towards stroke recovery. These cargo, likely, are key mediators providing paracrine benefits in brain. As the regenerative potential of MSC-EVs is frequently investigated for a plethora of different conditions, we have summarized all known cargo and beneficial factors below [70,71,72,73,74].

### 5.1. Proteins, Growth Factors, and Cytokines

MSCs exposed to ischemic mouse brain tissue, both in vitro and after experimental stroke in vivo show significant upregulation of beneficial growth factors secreted through EVs. The factors released by MSCs include vascular endothelial growth factor-A (VEGF-A), VEGF-C, fibroblast growth factor 2 (FGF2; also basic FGF or bFGF), placental growth factor (PGF), hepatocyte growth factor (HGF), and interleukin (IL)-6, among others [75,76,77,78], many of whom are carried by EVs [78]. Additional proteins found in MSC-EVs also include Angiopoietin 1, Notch 2, vascular cell adhesion molecule 1 (VCAM-1), and transforming growth factor-β2 (TGF-β2) [78]. These molecules promote survival, neuroprotection, and promote angiogenesis in damaged tissue.

### 5.2. miRNAs

MicroRNA (miR) are small, endogenous, non-coding RNA molecules with the ability to selectively hybridize to the 3′-UTR poly(A) tail of targeted mRNAs, blocking their transcription into proteins or enhancing their degradation [79]. It has been suggested that the effects of EVs come mainly from miRNAs [70,80], though this may be because it is the most studied EV cargo. miRNAs can be carried inside the EVs and transported to brain to provide pro-regenerative effects after stroke. Indeed, pre-treatment of MSC-EVs with RNase impaired the ability of these EVs to provide the protective effects to injured tissues [81].

### 5.3. Other Factors Carried by EVs

The majority of EV research has focused on miRNA, but other RNA species that can be found in EVs include messenger RNA (mRNA), long non-coding RNA (lncRNA), ribosomal RNA (rRNA), transfer RNA (tRNA), RNA repeats, and circular RNA (circRNA) [82,83,84,85,86]. EV cargo can also include DNA and other genetic materials that reflect their cellular origin [87,88]. Most research so far has focused on the effects imparted by transfer of proteins and miRNAs, with little emphasis on the other potential cargos.

MSC-EVs have been shown to transfer mitochondria and mitochondrial DNA (mtDNA) to injured cells [89,90,91]. In vitro studies show mitochondrial transfer is able to rescue damaged ECs from ischemia/reperfusion injury, though the precise mechanism is unknown [91,92]. Mitochondrial dysfunction in the infarct and peri-infarct areas is one of the hallmarks of ischemic injuries and directly contributes to its pathophysiology [93]. Due to lack of blood flow, these organelles, known as the powerhouse of the cell, are unable to keep producing the necessary ATP for proper cellular functions. Without adequate oxygen, mitochondria produce high levels of ROS that causes oxidative damage and hypoxic-inducible factor (HIF), the main factor driving hypoxia-induced gene and protein changes [94,95]. Under hypoxic conditions, the increase in ROS and decrease of 2-oxoglutarate and oxygen inhibit the activity of prolyl hydroxylases (PHD), the enzymes responsible for hydroxylation and degradation of HIF under normoxia [94]. This inhibition of PHD allows the stabilization of HIF which then translocates to the nucleus where it affects specific gene expression [94]. Eventually, the dysfunctional mitochondria undergo mitophagy and release factors such as cytochrome c which leads to the onset of cellular apoptosis [96]. Targeting mitochondrial repair has recently been suggested as a new avenue for ischemic stroke treatment [93,97,98].

Lipids are another notable cargo transferred by EVs. In fact, the lipid makeup of the vesicle membrane can be used to determine the type of EV. Exosomal membranes appear to be enriched in glycolipids and free fatty acids, while the MV membranes are enriched with ceramide and sphingomyelins [54]. EV’s bioactive lipid cargo include eicosanoids and related metabolic enzymes [99,100]. Lipid components of the EVs has not been investigated as frequently as nucleic acids and protein components, though there is a lot that can be learned through furthering its research [99].

Publicly available online databases such as ExoCarta (exocarta.org) (accessed on 28 March 2021) and Vesiclepedia (microvesicles.org) (accessed on 28 March 2021) have been created to manually document the known molecules identified within extracellular vesicles. ExoCarta collects information regarding the cargoes of exosomes, whereas Vesiclepedia includes microvesicles and apoptotic bodies in their consortium [101,102]. As of 2019, Vesiclepedia has compiled over 1200 studies cataloging the presence of about 32,000 unique proteins, 17,000 unique mRNAs, 2400 unique miRNAs, and more than 600 lipids and metabolites [101].

## 6. Effect of MSC Derived Extracellular Vesicles on Neuro-Vascular Unit after Stroke

**Figure 1:**
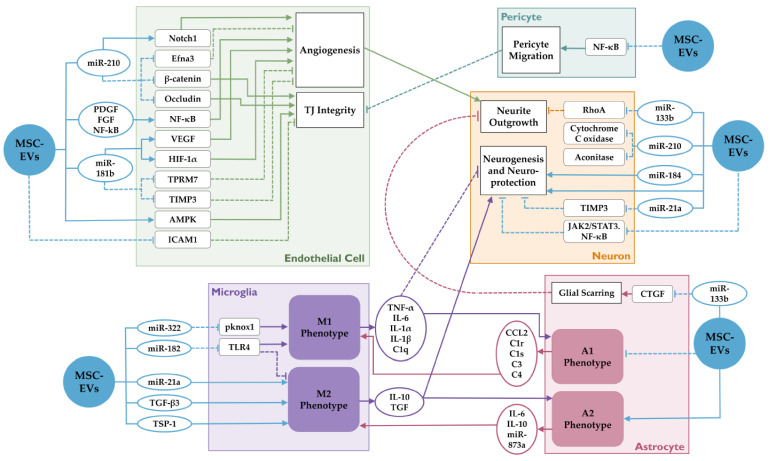
Molecular effects of MSC-EVs on various cells in the neurovascular unit (NVU) in stroke. MSC-EVs and its contents affect diverse molecular pathways in endothelial cells, microglia, astrocytes, neurons and pericytes. They regulate various processes such as angiogenesis, tight junction integrity, pericyte migration, neurite outgrowth, neurogenesis, and microgial polarization. These complex interactions between different cell types determine the neurological outcome after stroke. Dotted lines indicate inhibition, while solid lines indicate potentiation.

MSC-EVs elicit a diverse set of molecular changes in recipient cells based on their content and cell type. These changes may affect processes that aid in stroke recovery. Figure 1 outlines some of the effects of MSC-EV’s on NVU cells.

### 6.1. Endothelial Cells

One of the key factors contributing to increased BBB permeability after stroke is MMP-9. Immediately after stroke onset, neutrophils migrate and adhere to the brain endothelium through intracellular adhesion molecule 1 (ICAM-1), where they release a large amount of MMP-9 that degrades the basal lamina and TJs holding ECs together. TJ protein, Claudin-5, has been investigated as a promising target to allow selective diffusion of drugs across the BBB [103]. In fact, claudin-5 deficient mice do not show increased breakdown of the BBB or vascular dysfunction, however the permeability of the BBB is altered to allow increased paracellular transportation to molecules smaller than 800D [103]. MMP-9 overexpression in brain ECs can result in significant degradation of claudin-5 [104]. MSCs can prevent this degradation, since it is known that they induce phosphorylation of AMP-activated kinase (AMPK), and suppress the expression of ICAM-1 [105].

miR-210 is induced by hypoxia in every cell type investigated so far, including MSCs, although it has both beneficial and detrimental effects on the BBB. MSC-EVs have been found to contain miR-210, which can be internalized by endothelial cells [106]. Through downregulation of Efna3, a GPI-anchored membrane protein, miR-210 promotes angiogenesis [106]. Furthermore, overexpression of miR-210 in endothelial cells enhances Notch1 signaling and shows increased migration on functional assays using matrigel, indicating a possible positive role of miR-210 in angiogenesis [107]. On the other hand, miR-210 has also been shown to increase BBB leakage and cerebral edema by downregulating expression of occludin and β-catenin in neonatal rat hypoxic-ischemic brain injury model [108]. These conflicting findings regarding the effect of miR-210 on BBB integrity indicates the need for further studies to ascertain their role in regulating BBB function. 

Angiogenesis and increasing microvessel density following ischemic stroke is associated with neural remodeling and recovery [57]. Blood vessels may serve as a scaffold to guide newly formed neurons and axons, and functional vessels deliver much needed nutrients and oxygen to supply endogenous repair mechanisms [57,109,110,111]. The main angiogenic effects of MSC-EVs are believed to be mediated through activation of the NF-κB pathway [112,113]. Platelet-derived growth factor (PDGF), FGF, and epidermal growth factor (EGF) have been identified in the MSC secretome to induce phosphorylation of NK-κB [112,113]. Exosomes from adipose derived stem cells contain miR-181b, which contribute to in vitro angiogenesis of brain microvasculature endothelial cell (BMEC) after oxygen-glucose deprivation (OGD) [114]. This is achieved by downregulating the expression of transient receptor potential melastatin 7 (TRPM7), as well as reducing expression of TIMP3 and upregulating the expression of HIF-1α and VEGF [114]. Other researchers have shown that silencing TRPM7 promotes cellular migration and tube formation [115].

### 6.2. Astrocytes

Astrocytes are the 2nd most common glial cell type in the brain and they have a plethora of functions in maintaining a healthy and functional environment [116]. As a part of the NVU, astrocytic end-feet processes ensheath the vasculature and regulate blood flow [43]. They can also act as CNS immune cells and play a role in neuroinflammation and regeneration [117,118]. Following injury, astrocytes undergo a transformation known as “reactive astrocytosis” whereby they activate. Similar to the classification of activated microglia, there are two types of activated astrocytes; pro-inflammatory “A1” astrocytes and anti-inflammatory “A2” astrocytes—although this nomenclature is not widely used [117,119]. These activations coincide with other immune cell activity, especially microglia, making it a challenge to differentiate each of their specific contributions to neuroinflammation [117].

Proliferation of activated astrocytes forms a glial scar that physically and chemically inhibits neuronal outgrowth [120]. Administration of MSC-EVs could help reduce glial scarring through transfer of miR-133b which, in astrocytes, reduces the expression of connective tissue growth factor (CTGF), a peptide belonging to a family of extracellular matrix (ECM)-associated proteins involved in intracellular signaling [68]. Additionally, A1 astrocytes have demonstrated neurotoxic properties [121]. On the other hand, astrocytes activated by IL-1β can release factors that promote neuronal growth and angiogenesis, including the damage-associated molecular-pattern molecule high-mobility-group-box-1 (HMGB1), which increases migration of endothelial progenitor cells (EPCs) to cerebral vessels where they take part in angiogenesis and functional recovery [122]. Furthermore, astrocytes can transfer functional mitochondria to injured neurons to increase cell survivability [123].

Astrocytic activation is mediated by intracellular communications with microglia [121,124,125,126,127]. When cultured alone, astrocytes produce only a very small response to lipopolysaccharides (LPS), a commonly used substance to induce inflammation [124]. Microglial secretions of C1q, IL-1α, and tumor necrosis factor (TNF) are able to induce A1 astrocytes, and the contents of microglia-EVs modulate the activation state and secretions of the recipient astrocytes [121,126,128,129]. In turn, the secretions of astrocytes reciprocally regulate microglial phenotype [119,129,130]. Administrating microvesicles collected from ECs in vitro can communicate with astrocytes to help prevent ischemic brain injury in vivo by regulating their proliferation and apoptosis [131]. Astrocytic EVs could also contribute to neural plasticity during stroke recovery. In vitro experiments suggest that some of the neuroregenerative effects of MSC-EVs result from secondary EV release from astrocytes [132]. Further studies are needed to ascertain whether the effects of activated astrocytes on neuronal survival and regeneration are beneficial or detrimental [117,125,133].

### 6.3. Pericytes

Pericytes are a key component in microvascular integrity and repairs following ischemic stroke. They are in close contact with capillary endothelial cells, which allows them to regulate blood flow and stabilize the structure of the vessels [134]. In addition, they have several similarities to MSCs including the same cell surface markers and multipotent stem cell activity [134]. Indeed, it has been suggested to substitute MSCs for pericytes when modeling the BBB in vitro, though this view is controversial and pericytes are commonly utilized, albeit with variable effects [135,136,137].

Research has shown that MSC-EVs can modulate pericytes. Using a model for spinal cord injury, administration of MSC-EVs was shown to reduce pericyte migration from the region of blood-spinal cord barrier damage via downregulation of NF-kB p65 signaling [138]. Growth factors in MSC-EVs impart beneficial effects on maintaining the BBB integrity via pericytes. For example, in ischemic conditions, VEGF receptor 1 (VEGFR-1) is upregulated on pericytes [139]. Intranasal administration of VEGF-B starting 24 h after stroke for 3 days enhances the survival of pericytes and increases their association with microvascular endothelial cells, resulting in enhanced microvascular stability and BBB integrity [139]. In contrast to the pro-angiogenic properties of VEGF-A that increase BBB permeability, VEGF-B acts as a pro-survival factor that decreases vascular leakage and increases the number of microvessels within the ischemic tissue [139]. However, currently there is little information on the interactions between pericytes and MSC-EVs. Further research focusing on pericytes stabilizing the BBB after ischemic injury is needed.

### 6.4. Microglia and Macrophages

Microglia, the resident macrophages of the CNS, can be activated into the “classical” pro-inflammatory M1 and “alternative” neuroprotective M2 phenotypes in response to microenvironmental signals [140,141,142,143,144]. M1 macrophages secrete a high amount of inflammatory cytokines such as TNF-α, IL-1β, and IL-12; whereas M2 macrophages secrete high amounts of anti-inflammatory and neuro-protective cytokines such as IL-4, and IL-10. During the early, acute phase of ischemic stroke, microglia and recruited macrophages develop the anti-inflammatory, neuroprotective M2 phenotype, protecting the neurons in the infarct area, phagocytizing cellular debris resulting from ischemic injuries, and helping to restrict the area of damage [145,146,147]. As the stroke progresses over the next several days, M1 microglia and macrophages become more numerous [145,148]. Phagocytosis of dead cells and debris decreases, especially in macrophages, and the neuronal damage caused by ischemia is increased [142,145,149]. M1 microglia tend to contribute to NVU disruption, while M2 microglia are considered restorative [144,150]. Returning these cells to their anti-inflammatory profile during the subacute and chronic phases of stroke may reduce the extent of BBB permeability and promote its recovery after ischemic incidents.

Administration of MSCs has been shown to induce M2 polarization through various pathways [106,151,152,153,154,155,156,157]. MSC exosomes contain miR-322, which suppresses the expression of pknox1, a protein that encourages the M1 phenotype, thus promoting M2 [157]. Other various factors in the MSC secretome directing M2 polarization include Tissue growth factor-β3 (TGF-β3) and Thrombospondin-1 (TSP-1) [152]. Enzymatically active membrane particles generated by MSCs are able to selectively bind to pro-inflammatory monocytes and induce their apoptosis, thereby reducing the amount of inflammatory cytokines such as TNF-α and IL-12 released in the injured tissues [154]. MSC-EVs containing miR-182 negatively regulate the toll like receptor 4 (TLR4)/ NF-κB pathway in macrophages, encouraging M2 polarization [155]. In a hypoxia-ischemic injury model in neonatal mice, miR-21a was transferred to microglia via MSC-EVs where it induced anti-inflammatory M2 polarization and enhanced neuroprotection [156]. Through the promotion of anti-inflammatory microglia and macrophages phenotypes, MSC-EVs reduce neuroinflammation to foster an environment facilitating recovery [153,158].

### 6.5. Neurons

Restoration of the NVU is required for brain parenchymal recovery. Aside from supplying oxygen and nutrients vital to neurons, the cerebral vasculature acts as a scaffolding to guide the migration of axons and newly formed neurons [57,109,110,111]. MSC-EVs have been reported to provide beneficial effect on neurons through various mechanisms. miR-210, which can be delivered via MSC-EVs, show various effects on neurogenesis following brain injury, possibly due to differences in time of administration [151,159,160,161]. Inflammatory factors IL-6 and TNF-α secreted by microglia after injury have been shown to decrease the survival of Doublecortin-positive (Dcx+) cells in vitro [159]. Dcx, often used as a marker for neurogenesis, is expressed by actively proliferating neuronal precursor cells and immature, migrating neuroblasts [162,163]. miR-210 inhibition increases the survival of Dcx+ cells by eliminating the harmful effect of IL-6 and TNF-α [159]. On the other hand, Cytochrome C Oxidase and Aconitase are downregulated by miR-210 [159]. Although miR-210 has neuroprotective effects, inhibiting it too early reduces neural stem cell proliferation and glycolytic activity, and it has been suggested to delay miR-210-related treatments until after the peak proliferation period [159]. More research is needed to investigate a balance between the beneficial and deleterious effects of miR-210 treatments on the overall NVU.

MSC-EVs have been shown to regulate the AMPK and JAK2/STAT3/NF-κB signaling pathways [164]. In a rat stroke model, MSC-EVs significantly alleviated neurological deficits by increasing phosphorylation of AMPK, and reducing phosphorylation of JAK2, STAT3 and NF-κB [164]. Following MCAO and MSC treatment, exosomes containing miR-133b have been detected in neurons where they promote neurite outgrowth via down-regulation of RhoA expression [68]. MSC-EVs containing miR-184 have also been shown to promote neurogenesis [165]. In vitro imaging shows that internalization of MSC exosomes speeds up axonal growth of cortical neurons and reverses the inhibitory effects of chondroitin sulfate proteoglycan (CSPG) expressed by glia after injury [166]. MSC-EV transfer of miR-21a to neurons enhanced neuroprotection by targeting Timp3 [156]. These studies underscore the importance of MSC-EV cargoes in promoting the health of neurons.

## 7. Methods to Enhance Therapeutic Potential of MSC Derived Extracellular Vesicles

Cumulative evidence from many studies described thus-far suggest that MSC-EVs carry beneficial cargo which can aid in the repair of NVU. To make the MSC-EV therapy more effective, future studies must focus on enhancing this therapeutic potential of MSC-EVs. Some of the strategies to enhance this potential are discussed below.

### 7.1. Engineering MSCs to Generate EVs with Beneficial Cargo

#### 7.1.1. Use of Viral Vectors to Modify MSCs

The most effective way to attain modified MSC-EVs is to culture modified MSCs and collect their EVs. The expression profile of the MSC secretome can be altered by changing the MSCs via viral vectors; most notably adeno-associated virus (AAV), adenovirus, retrovirus, and lentivirus. Although not many studies have focused on using the MSC-EVs from modified MSCs, there is ample evidence showing benefits of genetically modifying MSCs in ischemic stroke [167,168,169,170].

MSCs transfected by lentivirus to overexpress brain-derived neurotrophic factor (BDNF) and VEGF-A have greater neuroprotective effects when transplanted to rats 2 h after global cerebral ischemia injury [167]. One week after ischemia, the rats who received BDNF and VEGF-overexpressing MSCs exhibited increased functional recovery with less cell death as evidenced by significantly reduced TUNEL-positive cells and brain edema [167]. MSCs engineered to overexpress VEGF and Ang-1 via adenovirus showed higher stable neovascularization and a larger reduction in lesion volume than MSCs overexpressing each individually [170]. Adeno-associated virus-mediated overexpression of IL-10 in the secretions of MSCs administered following middle cerebral artery occlusion (MCAO) in rats reduced infarction volume by 39% compared to control after 3 h [168]. Further, there was a substantial reduction in activated Iba-1-positive microglia and pro-inflammatory cytokines present in the brain tissue during the acute phase [168]. MSCs transfected with erythropoietin (EPO) increased the expression of BDNF, platelet-derived endothelial cell growth factor (PD-ECGF), HGF, stromal cell-derived factor-1a (SDF-1a), and TGF-1β [169]. Future studies must utilize these techniques to improve the delivery of beneficial factors to the NVU via EVs.

#### 7.1.2. Crispr-Cas9 System to Modify MSCs

Viral and non-viral delivery systems for clustered, regularly interspaced, short palindromic repeats (CRISPR)-CRISPR associated protein 9 (Cas9) techniques allow precise genome editing with high efficiency and low off-target interactions [171,172]. CRISPR-Cas9 allows transfection of large transgene cassettes that may otherwise be too large for other techniques [173]. One study used CRISPR-Cas9 system to enhance MSC secretion of soluble Receptor for Advanced Glycation End-products (sRAGE) proteins hoping to decrease AGE-RAGE-induced neuronal apoptosis in a model for Parkinson’s disease [174]. Though they only tested sRAGE-MSCs on their first passage after transfection, their results showed that the protein secreted by the modified MSCs lasted a longer time than administration of the protein itself or non-modified MSCs [174]. However, secretion of sRAGE decreased in subsequent passages due to low transgene integration, despite high transfection rates [174]. More research needs to be done on increasing transgene integration and protein secretion in subsequent passages to be able to scale up this production method before it can be fully utilized. CRISPR-Cas9 systems are also being investigated for reversible repression of specific gene transcription, optogenetic control of gene editing, and targeting RNAs [172,175,176,177,178,179], opening up new doors for investigating the cellular mechanisms responsible for stroke pathology and treatment.

#### 7.1.3. Non-Viral Methods to Modify MSCs

Physical, non-viral methods such as nano-injection, cell-penetrating peptides, ultrasound irradiation, and electro-permeabilization have been used for gene transfer into cells, though these methods all have their pros and cons. While these methods are considered safer and mildly less controversial for use in human therapies, potential disadvantages for non-viral gene transfer include tissue or cell damage, low transfection efficiency, high cell mortality, and being time consuming [172,180]. Currently non-viral methods are utilized less frequently than viral modifications, though this is a growing field with potential to broaden the prospects of genomic-based therapies.

Electroporation-mediated gene transfer of growth differentiation factor 5 (GDF5) into MSCs resulted in gene expression similar to those produced by an adenovirus vector for the same gene for up to 3 weeks [181]. A study used microporation, a modified electroporation technique, to transplant BDNF and enhanced green fluorescent protein (EGFP) pDNA into MSCs, which promoted their in vitro differentiation into neural cells without affecting the key properties of MSCs such as their proliferative abilities and immunophenotype [182]. By using EGFP as a reporter to monitor transfection efficiency, researchers found 83% efficiency using a microporation device while standard electroporation devices had 30–50% efficiency [182].

Nanoparticles such as mesoporous silica nanoparticles (MSNs) carrying genetic material can be loaded into MSCs to use as a vehicle for targeted delivery to a specific location. One study aiming to increase the endocytosis of the MSNs coated them with a hyaluronic acid-based polymer (HA) allowing them to bind to CD44 receptor for uptake. Despite this, most of the MSN uptake was dependent on phagocytosis and pinocytosis, however the HA coated MSNs exhibited a higher retention capacity up to 3 weeks later [183]. Aminated MSNs (MSN-NH_2_) complexed with pDNA increase the uptake into MSCs, with an approximately 68% transfection efficiency [184]. More investigation into MSC-EV-mediated delivery of nanoparticles is warranted as nanoparticles are small enough to pass the intact BBB and therefore present a promising method for delivering therapeutics.

Cell penetrating peptides (CPP; or protein transduction domain, PTD) are facially amphiphilic complexes that can be used to efficiently transfer genetic information or macromolecules through a cell’s plasma membrane. Positively charged micelles are formed which interact with negative charged molecules such as nucleic acid to form nano sized conjugates [185,186,187]. In an experiment testing the three most representative bile acid-modified polyethyleneimine (BA-PEI) to transfer VEGF pDNA into MSCs, the deoxycholic acid (DA-PEI) conjugates were more successful than cholic acid (CA-PEI) conjugates and lithocholic acid (LA-PEI) conjugates for gene transfection and overexpression [185]. Alternatively, gold nanoparticles (AuNPs) have frequently been conjugated to PEI (Au-PEI) to act as gene vectors for their high transfection efficiency as well as their strong fluorescence, granting the ability to track the particles in vivo using a bioimaging system [188,189,190]. The safety and efficacy of CPP therapy has been evaluated in numerous preclinical studies, and some methods have moved on to clinical trials [191]. Cell penetrating peptides have been used to deliver a variety of cargoes, including peptides, pDNA, dsDNA, siRNA, nanoparticles, liposomes, proteins, small drugs, and antibodies [185,187,191,192,193].

### 7.2. Pre-Exposure and Preconditioning of MSCs

#### 7.2.1. Hypoxic Preconditioning

Hypoxic preconditioning has been shown to alter the cargos of MSC-EVs. MSC incubation in 0.5% O_2_ for 24 h improved their resistance to apoptosis in nutrient-poor environments, possibly by increasing the phosphorylation of Akt and NF-κB p65 [194]. Preconditioning in 3% O_2_ was shown to increase the expression of C-X-C chemokine receptor type 4 (CXCR4) and CXCR7 proportional to length of exposure, until maximal levels are reached at 24 h [195].

In vitro hypoxic preconditioning of MSCs increases the expression and secretion of VEGF, SDF-1, CXCR4, BDNF, glial cell line-derived neurotrophic factor (GDNF), EPO, Angiogenin, insulin-like growth factor (IGF), and IL-6 [196,197]. In a rat model for traumatic brain injury, rats infused with hypoxia pre-conditioned MSC secretome showed less brain damage than those treated with the normoxic MSC secretome, though both hypoxic and normoxic groups displayed decreased incidence of brain damage (27% and 66%, respectively) compared to control (100%) [198]. Further, the rats treated with the hypoxic MSC secretome performed significantly better on motor and cognitive tests 4 days after TBI than those treated with the normoxic MSC secretome [198]. Table 2 shows published literature showing benefits of hypoxic-preconditioned MSCs in stroke recovery.

#### 7.2.2. Preconditioning with Growth Factors, Cytokines, and Other Compounds

Exposure to specific growth factors in culture also changes the content of MSC-EVs. It has been shown that culturing MSCs in endothelial differentiation medium (EDM) upregulated both the number of microvesicles released and their angiogenic effect on human umbilical vein endothelial cells (HUVECs) in vitro [207]. One factor significantly upregulated in the secretome of MSCs under EDM preconditioning was miR-31, a molecule which promotes migration and tube formation in HUVECs by suppressing FIH1, an asparaginyl hydroxylase enzyme that inhibits HIF-1 [207].

After exposure to interferon-γ (IFN-γ), indoleamine 2,3-dioxygenase (IDO) is upregulated in MSCs [208]. This upregulation has been shown to suppress T cell proliferation and influence monocyte differentiation into the M2 phase [208]. In addition to immunosuppression, IFN-γ-exposed MSCs have greater homing and regenerative properties [209]. Conditioning MSCs with pro-inflammatory cytokine IL-1α or IL-β increases the expression of granulocyte-colony stimulating factor (G-CSF) [210]. When the CM of pro-inflammatory factor-primed MSCs were added to immortalized mouse microglial BV2 cells in vitro, it induced secretion of anti-inflammatory cytokines such as IL-10 and decreased the secretion of pro-inflammatory factors [210]. MSCs primed with FGF-2 increased angiogenesis in vivo through increased secretion of VEGF and HGF [211]. Culturing MSCs in a NO-releasing polymer is able to increase the angiogenic effects of its EVs [212]. Increased levels of miR-126 were found both in the MSCs and in their released exosomes [212]. miR-126 decreases the expression of PIK3R2 in ECs, thus enhancing growth factor signaling and promoting the pro-angiogenic PI3K/Akt pathway [212]. MSCs with 2 h exposure to 200 μmol/L H_2_O_2_ secreted approximately 25-fold higher amounts of IL-6 in the conditioned medium, though the secretion of VEGF and TNF-α remained unchanged [213]. Expanding MSCs with serum from ischemic stroke patients instead of fetal bovine serum (FBS) resulted in increased cell proliferation associated with enhanced expression of miR-20a which silences p21 (also known as CDKN1A) [199]. These findings from various published studies show that MSC secretome can be changed to generate more beneficial EVs.

#### 7.2.3. 3D Cultures

In addition to manipulating the media, other culture conditions can also affect MSC cargo, such as 3D conditions. Administration of EVs derived from MSCs cultured in 3D and 2D conditions 24 h after traumatic brain injury in rats increased the amount of new endothelial cells and reduced inflammation [214]. The number of EVs produced by MSCs in 3D conditions was significantly greater than those in 2D conditions and led to increased functional recovery [214]. Culturing MSCs in 3D conditions better imitates in vivo, physiological conditions and produces spheroidal cell aggregates which exhibit increased survivability and enhanced expression of anti-inflammatory, angiogenic, and regenerative genes and proteins [215,216,217]. Genes encoding for Ang-2, ANGPT2, CXC4, IL-1α, IL-1β, IL-6, IL-8, TGFβ-1, FGF2, and VEGF were upregulated in 3D cultures of MSCs [216,217]. EVs derived from 3D MSC cultures could have significantly higher regenerative properties, which need to be studied in the further detail.

## 8. Improved Homing for Targeted EV Delivery

Similar to MSCs getting stuck in the lungs and liver which prevents them from reaching the area of injury [218,219,220,221], many intravenously administered exosomes are still caught by the liver until being excreted through the bowels [222]. However, MSC-EVs have better homing to the ischemic brain areas where they are taken up by local cells, peaking on day 3 but remaining for up to 14 days [222]. MSC-derived exosomes were shown to have the same immunosuppressive and reparative properties as their parent cells [222,223].

The uptake of EVs by target cells is influenced by the surface receptors and ligands present on their outer membranes [224]. The tetraspanin Tspan8 has been shown to recruit cargoes such as CD49d into EVs that promote angiogenesis once internalized by ECs [225]. A study was able to demonstrate targeted delivery of EV cargo to brain cells by engineering cells to express a membrane protein complex containing Lamp2b and CNS-specific rabies viral glycoprotein (RVG) peptides [226]. Loading these EVs with GAPDH siRNA through electroporation successfully caused gene knockdown in CNS cells with little non-specific uptake in other tissues [226]. Though more investigation is needed to further expand this area, transfecting MSC-EVs to express homing molecules for specific cell types is a large step towards tailored drug delivery systems.

Long-term research should focus on the combination of three factors: (1) Enhancing homing abilities of MSCs and MSCs-EVs; (2) Selective targeting of MSCs for specific transgene expression; and (3) the customization of MSC-EV cargoes, as this could open up new avenues for efficient drug delivery across a previously impermeable BBB.

## 9. Future Directions, Considerations, and Conclusions

Further research is needed to elucidate more mechanisms and underlying pathways responsible for the therapeutic effect imparted by the EVs secreted by MSCs. Clinical trials using MSCs have had variable results, some showing beneficial effects, while others fail to find significant therapeutic benefits [227]. Some of the variation in results may be attributed to the heterogeneity of MSCs, a factor that is difficult to control for and coordinate across studies [227]. Factors such as age of donor, tissue type harvested, isolation protocol, dosage, and passage number all affect the heterogeneity of MSCs and patient response [227,228]. Even within a single project, MSCs collected from different donors were shown to possess variable immunosuppressive potential [208]. Furthermore, many clinical trials are performed using a small number of patients, sometimes without proper controls or a standard protocol [227]. An additional hurdle in translating the preclinical results to effective treatments in humans is the physiological and genetic differences between species.

Animal models of human ischemic stroke are limited by how much preclinical research may be accurately applied to clinical translations. Unlike animal models, human patients may have comorbidities and underlying health problems that may impact the pathophysiology of stroke and must be taken into account when determining the most appropriate treatments, though rodent strains may be available to model one underlying condition at a time. Further, although the genome is largely conserved between mammals, differences in gene and protein expression may impact fundamental processes such as inflammation and immune response [229]. Humanized mice can be used to overcome some of the intrinsic differences, though they are not widely utilized. Further research aimed at identifying and understanding the human-specific genes involved in stroke may lead to better animal models and treatment targets [229].

Future emphasis must be placed on repairs and regeneration of the NVU, as it is essential for brain health and homeostasis. Even though an intact BBB may block certain drugs and molecules from reaching the brain, the expansion of new and developing molecular techniques may soon render a feasible method to bioengineer a backdoor. The future of drug delivery into the CNS starts with customizing and bioengineering EVs. With improved homing, EVs can target injured endothelial cells or junction proteins on the BBB. Research is on the right track to achieve delivery of specialized receptor ligands that could selectively manipulate the permeability of the BBB. Until then, the techniques for modifying and customizing EV contents and delivery should be emphasized and researched in greater detail. Expanding the capabilities of such methods may lead to further insights on mechanisms, cascades, and cellular pathways involved in each step of ischemic stroke.

## Figures and Tables

**Table 1:** Subtypes and Properties of Extracellular Vesicles.

	Exosomes	Microvesicles	Apoptotic Bodies	Oncosomes
Alternative Names	Small EVs	Ectosomes, Shedding vesicles, Microparticles, Exovesicles		Large Oncosomes
Intracellular Origin	Multivesicular Endosome	Plasma Membrane	Membrane blebbing during cell death	Non-apoptotic tumor-cell membrane blebbing
Size	50–150 nm	150–1000 nm	100 nm–5 um	1 um–10 um
Differential Ultracentrifugation	100,000× *g* for 90 min	10,000× *g* for 30 min	800× *g* for 10 min; then 16,000× *g* for 20 min	Alternate 8000× *g* for 30 sec and 0.2 µm filtration; Oncosomes are caught by filter
Enriched Protein Pathways	Extracellular matrix; Heparin-binding; receptors; Immune response; Cell adhesion	Endoplasmic reticulum; Proteasome; Mitochondria	Heterogeneous	Extracellular matrix degradation; Angiogenesis; Cancer metabolism
Enriched Lipid Contents	Glycolipids, Free fatty acids, Phosphatidylserines	Ceramides and Sphingomyelins		
Structural Plasma Membrane Lipids	Phosphatidylserine enrichment; Phosphatidylcholine, Phosphatidylglycerol, Phosphatidylinositol, and Phosphatidylethanolamine depletions	Dependent upon cellular origin; Most have phosphatidylglycerol, phosphatidylinositol, and phosphatidylethanolamine depletions	Phosphatidylserine enrichment	Phospholipid and phosphatidylserine enrichment
Contents	Proteins, Lipids, RNAs	Organelles, Proteins, Lipids, RNAs	Organelles, Histones, DNAs, RNAs, Nuclear fractions	Proteins, RNAs

**Table 2:** In Vitro and In Vivo Effects of Hypoxia-preconditioned MSCs in Stroke Recovery.

Condition	Cell Type	Secretome Changes	In Vitro Effects	In Vivo Effects	Source
Hypoxia	Rat BMSC	Increased HIF-1a, VEGF, FIK-1 SDF-1, CXCR4, BDNF, GDNF, EPO, EPOR, Angiotensin-1; Decreased Complement C3 and C5, IL-1α, L tb, Tnfrsf1a, Tnfrsf1b	-	Higher vessel density in ischemic core and penumbra; Stronger inhibition of OX-42+ microglia; Increased NeuN+ cells; Significant increase in motor function recovery;	[196]
Stroke Serum	Rat BMSC	Increased miR-20a	Enhanced MSC proliferation	-	[199]
Hypoxia (CoCl_2_)	Human MSC	Increased miR-124a, HIF-1α, DCX, Tuj1,	Increased neuronal differentiation	-	[200]
Ischemic Brain extract	Human MSC	Increased BDNF, VEGF, and HGF	-	-	[201]
Hypoxia	Rat BMSC	-	-	Reduced infarct volume; Decreased extravascular leakage; Increased angiogenesis and improved blood flow; Decreased behavioral deficits;	[202]
MCAO Rat Brain Extract	Rat AD-MSC	Increased miR-212, miR-181b;	Increased OGD-BMEC migration and angiogenesis; Increased BMEC expression of HIF-1α, VEGF; Decreased BMEC expression of TIMP-3	-	[115]
Hypoxia	hUCB-MSC	Increased Thrombospondin1, Pantraxin3, VEGF	-	-	[203]
Hypoxia	Rat BMSC	-	-	Increased migration of MSCs to infarct region possibly due to upregulation of CXCR4, MMP-2, and MMP-9; Reduced brain infarct volume and cell death; Attenuated neurological deficits;	[204]
Hypoxia	hUCB-MSC	Increased VEGF, Angiogenin, IGF, IL-6, Tie-2/TEK, UPAR	-	-	[197]
Hypoxia	Rat BMSC	-	Increased proliferation and migration possibly due to activation of PI3K/AKT pathway	Reduced cerebral inflammation and edema; Increased reduction of TNFα and S100B;	[205]
Hypoxia	Aged human BMSC	Increased VEGF	Increased viability of OGD neurons	-	[206]

## Data Availability

Not applicable.
